# Scale for the assessment and rating of ataxia: a live e-version

**DOI:** 10.1007/s00415-025-13071-7

**Published:** 2025-04-10

**Authors:** Avigail Lithwick Algon, Penina Ponger, Leonardo Daniel, Yael De Picciotto, Eran Gazit, Marina Brozgol, Jeffrey M. Hausdorff, William Saban

**Affiliations:** 1 Center for Accessible Neuropsychology and Sagol School of Neuroscience, Tel Aviv University, 69978 Tel Aviv, Israel; 2 Department of Occupational Therapy, Faculty of Medical and Health Sciences, Tel Aviv University, 69978 Tel Aviv, Israel; 3 Movement Disorders Division, Department of Neurology, Tel Aviv Sourasky Medical Center, 69978 Tel Aviv, Israel; 4 Center for the Study of Movement, Cognition, and Mobility, Neurological Institute, Tel Aviv Sourasky Medical Center, Tel Aviv, Israel; 5 Dept of Physical Therapy, Faculty of Medical & Health Sciences and Sagol School of Neuroscience, Tel Aviv University, Tel Aviv, Israel; 6 Department of Orthopaedic Surgery, Rush Alzheimer’s Disease Center, Rush University Medical Center and Rush Medical College, Chicago, USA

**Keywords:** Online, Cerebellar ataxia, SARA, Ataxia evaluation, Cerebellum, Video conferencing

## Abstract

**Background:**

Measuring ataxia severity is primarily conducted in-person using tests such as the Scale for the Assessment and Rating of Ataxia (SARA). However, given the motor and cognitive impairments of people with cerebellar ataxia (PwA), there are major limitations in ensuring the assessment is accessible and scalable. We aimed to develop and validate a novel test, enabling the remote assessment of ataxia severity, SARA-Le (SARA Live e-version).

**Methods:**

SARA-Le is a structured step-by-step test for administering the SARA through video conferencing. In two experiments, we administered SARA-Le to 106 PwA. In Experiment 1 (*n* = 23), we assessed concurrent validity by comparing SARA-Le and in-person SARA scores administered by an independent neurologist. In addition, we evaluated associations between nine gait measures and both SARA and SARA-Le scores. In Experiment 2 (*n* = 83), we assessed the efficacy, internal consistency, and correlations between SARA-Le and other related measures.

**Results:**

First, we found a high correlation (*r* = 0.89, *P* = 0.001) between SARA-Le and in-person SARA scores, supporting convergent validity. Second, SARA-Le and SARA scores were both similarly associated with the nine gait measures, supporting construct validity. Third, SARA-Le’s Cronbach’s alpha was very high (0.831), supporting internal consistency. Fourth, SARA-Le scores exhibited a positive correlation with disease duration (*r* = 0.44, *P* < 0.001), and a negative correlation with MoCA scores (*r* = − 0.27, *P* = 0.007), supporting construct validity.

**Conclusions:**

SARA-Le can serve as a remote technology-based protocol, improving the accessibility and scalability of ataxia severity evaluation.

## Introduction

Ataxia is characterized by disturbances in balance, gait, and coordination [1]. However, there are major limitations in assessing the severity of ataxia in an accessible and scalable manner, which can be crucial for clinicians, researchers, and people with ataxia (PwA).

For over a century, measuring the severity of ataxia has mostly been conducted in-person, despite technological advances. However, PwA often experience motor impairments that restrict their ability to travel to the testing site, making in-person participation a deterrent in clinical trials [2]. Moreover, most of the testing and monitoring procedures are conducted by medical professionals, which often involve extensive appointment waiting times and high economic costs [3]. This issue is particularly challenging in remote areas where resources and medical services are limited.

In the case of rare conditions such as cerebellar ataxia (CA; [4]), traditional in-person motor testing faces exceptional accessibility and scalability challenges given the paucity of participants. Studies involving people with CA typically have small sample sizes, often with fewer than 15 participants. This limitation in sample size can affect the robustness and generalizability of research findings, highlighting the need for more scalable testing methods. This is particularly true given that CA is progressive, and there is thus a need for continuous accessible monitoring of symptoms.

The traditional method of ataxia severity assessment is conducted in-person by an expert, utilizing tools such as the Scale for the Assessment and Rating of Ataxia (SARA; [5]). The SARA is an extensively validated scale composed of eight items that measure various aspects of ataxia, including gait, stance, sitting, speech, and coordination [6, 7]. This scale is applied by trained examiners in both clinical and research environments worldwide [8]. However, this measure and others assessing ataxia severity mostly cannot be used in their current form to assess the severity of ataxia at home in an accessible and scalable manner (see Discussion for exceptions). Thus, it is important to develop new technology-based tools that allow for measuring the severity of ataxia remotely.

In addressing the challenges associated with traditional in-person ataxia testing, several studies have utilized technologies for ataxia assessment [9–11]. However, these tools, primarily based on video, did not present a test that can be easily administered remotely to a large sample of participants, independently and safely, and without the need for additional costly devices or medical professionals.

To address the need for remote assessment, we introduce SARA-Le, a new online test designed for the remote assessment of ataxia severity. SARA-Le, an acronym for SARA Live e-version, is a step-by-step and structured protocol for administering the SARA via video conferencing (VC). The primary objective of SARA-Le is to serve as an economical, technology-based protocol that can be easily administered to anyone with internet access and a webcam, with no requirement for a caregiver present while ensuring participant safety. In addition, SARA-Le can be administered without an expert neurologist.

In the current paper, we provide a detailed description of the SARA-Le test and examine its feasibility, efficacy, reliability, and validity. In Experiment 1, in a within-participant experimental design, we evaluated the criterion of SARA-Le by comparing it with the SARA scores given by an independent neurologist in-person. Furthermore, we compared the scores of the SARA-Le and the in-person SARA to gait measures collected in-person. In Experiment 2, we assessed the efficacy, internal consistency, and convergent and discriminant validity of SARA-Le by comparing it to other measures, such as disease duration and the Montreal Cognitive Assessment (MoCA; [12]). These analyses allow us to assess the usefulness and validity of the SARA-Le protocol for remote ataxia assessment.

## Methods

### Participants

All participants had a diagnosis of CA, were above the age of 18, were required to be able to understand and provide informed consent, and had access to an internet connection and a device capable of VC. Individuals with other neurological conditions (not CA), psychiatric conditions, learning disabilities, and severe visual or auditory impairments were not included in the study. Tel Aviv University and Tel Aviv Sourasky Medical Center Institutional Review Board Committees approved the protocol, and all participants provided informed consent before participating.

### Design

In Experiment 1, we compared the online SARA-Le through VC (i.e., Zoom) to an in-person SARA on 23 participants. The latter was administered randomly by an independent neurologist within 6 months of the SARA-Le administration, blinded to the SARA-Le scores. We do not expect the 6-month period to introduce significant variability, as CA progression is typically slower [13]. In addition, during the VC session, we administered a demographic and medical background questionnaire and the MoCA test [12, 14, 15]. To provide an additional aspect of construct validity, we also conducted a gait analysis on 17 participants. In-person SARA scores and gait measures were independently collected in the Ataxia Clinic, Department of Neurology and Center for the Study of Movement, Cognition, and Mobility, Tel Aviv Sourasky Medical Center. The participants walked on an instrumented walkway (Zeno Walkway, ProtoKinetics, Havertown, PA, USA, dimensions: length 10.97 m, width 0.61 m) at their self-selected speed. To ensure a steady-state gait pattern on the mat, patients started walking several steps before and after. Walking back and forth on the walkway constituted a single trial. The patients completed two consecutive trials. The steps taken on the walkway were recorded and analyzed, while those occurring outside the walkway were ignored. Sudden stops, steps taken off the walkway or to lean on adjacent walls for support or as part of a near-fall event were excluded (< 5%); a near-fall event was defined by observational analysis as a balance loss that would have resulted in a fall without corrective actions [16]. In Experiment 2, we administered the same demographic and medical background questionnaire, the MoCA test [12, 14, 15], and SARA-Le through VC to a larger cohort of 83 CA participants.

### Description of SARA-Le

The instructions and scoring of each item in the SARA-Le test correspond to the in-person SARA. To adapt the SARA to remote testing, a few modifications were made. Specifically, given that SARA-Le is administered remotely without in-person supervision, we modified the first three items on the SARA that might entail a risk of falls, and we modified the last item from a supine to a seated position. The gait, stance, and sitting items were decomposed into well-defined subcomponents and were administered online using a decision tree flowchart of binary yes or no questions. While these scores are based on the participant’s self-report, the step-by-step instructions allow for clarity on both the part of the participant and the administrator (see also [16]).

In particular, the decision tree is based on a simple yes–no flowchart scoring method. Thus, for each item experimenter or clinician can make a simple and clear binary decision. The development of the decision tree included four main steps. First, to ensure that we cover all the relevant content validity of each question, we defined the objective of each question. Second, we developed a set of yes/no questions that assessed the relevant content. Each question was developed in a way that was clear, concise, and unambiguous. Third, we established dependencies to determine how each question relies upon another. For example, if the participant responded “no” to the question “Are you able to walk with any support or independently?” the experimenter would skip all remaining questions concerning gait. Fourth, we assigned scores based on the final stage of the yes/no questions. The participants provided self-reports by verbally responding to structured questions regarding their typical performance in each task.

Before administering items five to eight in the SARA-Le, the experimenter shared their screen to demonstrate each task with a video recording. Scores were assigned based on the existing SARA scale, ranging from 0 to 4 for each task, with the score averaged over both sides. Thus, the total range of scores of the SARA-Le is comparable to the in-person SARA. The SARA-Le was administered and scored by trained research assistants. The research assistants received training from the main investigator. The SARA-Le protocol includes a step-by-step script that the research assistants were required to follow for each item. The research assistants were allowed to administer the SARA-Le after a practice trial with the lead investigator and his approval. The presence of a family member or caregiver was not required. No falls or injuries occurred during testing.

### Gait measures

Walking data from each trial were analyzed to evaluate gait parameters. We included spatial and temporal parameters to depict general gait patterns as well as parameters regarding pace, rhythm, and variability [17].

### Statistical analysis

The correlations were calculated using either Pearson’s *r* or Spearman rho (dependent on data distribution and relationship type) to investigate the relationship between our measures. We also calculated the association between SARA-Le scores and SARA scores using the Intraclass Correlation Coefficient (ICC). Fisher’s *z* test was utilized to compare the difference between two correlation coefficients in Experiment 2. Statistical analyses were performed using Rstudio (version 4.3.2; Rstudio Team, 2023) and JASP (version 0.18.2; JASP Team, 2023).

### Data availability

A detailed, step-by-step, description of the protocol is available at this online link (see URL: https://drive.google.com/file/d/1kldwZbjpSQcc42WlWu-6Utc1uSQgSajT/view?usp=sharing).

Anonymized patient data not published within this article will be made available by request from any qualified investigator.

## Results

### Participants

In Experiments 1–2, a total of 106 individuals with a previously established diagnosis of CA were tested. The participants’ demographic and medical information is presented below (Table 1). In Experiment 1, we collected data from 23 SCA3 participants. In Experiment 2, we collected data from 83 participants with CA. The participants had diverse known subtypes^1^ and 24 with CA of unknown etiology.

**Table 1:** Demographic, disease duration, and MoCA scores of the participants in both experiments. Mean [SD] Range

	*N*	Age	Years of education	Females (%)	Disease duration	MoCA
Experiment 1	23	51.2 [8.1] 34–67	14.4 [3] 11–22	52.17	5.4 [5.7] 1–19	24.6 [2.9] 19–29
Experiment 2	83	59.7 [11.2] 40–80	16.8 [2.2] 12–23	65.01	8.9 [8.6] 1–40	26.3 [2.4] 21–30

### Experiment 1

**Fig. 1:**
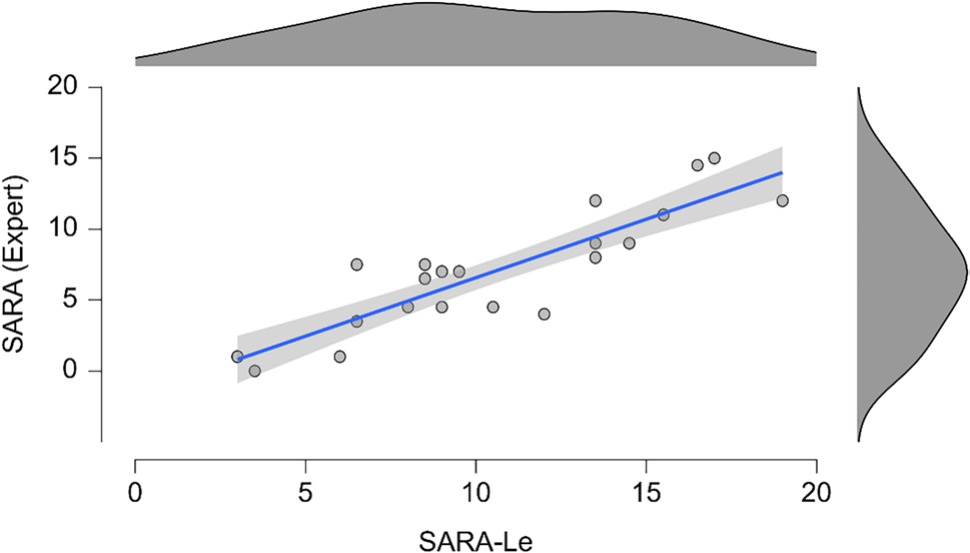
Correlation and trend line between SARA-Le scores and SARA scores

We found a very high positive correlation between the SARA-Le administered remotely and SARA scores provided by an independent neurologist administering the test in-person and blinded to the SARA-Le scores (*r* = 0.89, *P* < 0.001) see below (Fig. 1), supporting the convergent validity of SARA-Le. Additionally, The ICC between the SARA and the SARA-Le was 0.93 (95% CI [0.86–0.96]), supporting the reliability of SARA-Le. In addition, the sum of the self-report section (questions 1–3) of SARA-Le was strongly correlated with the sum of these items on SARA (*r* = 0.73, *P* < 0.001).

Furthermore, we observed significant correlations between the independent neurologist SARA scores and nine spatial–temporal gait measures (Table 2), which were collected in-person. Consistent with the results obtained through the neurologist’s in-person SARA scores, the SARA-Le scores exhibited significant correlations with the nine gait measures. All these correlations are of medium to high magnitude. Notably, in all gait measures, there was no difference between SARA and SARA-Le correlations (Fisher *Z* ranging from − 1.16 to 0.16; *P* > 0.05 for all correlations). For more information regarding gait measures, see below (Table 3).

**Table 2:** Correlations between the nine gait measures and either the in-person SARA or the SARA-Le scores

Measure	SARA		SARA-Le		Difference in correlation	
	*ρ*	*P*	*Ρ*	*P*	*Z*	*P*
Pace						
Gait speed (cm/sec)	− 0.49	0.029*	− 0.49	0.045*	− 0.01	0.992
Rhythm						
Cadence (steps/min.)	− 0.62	0.004*	− 0.58	0.015*	0.16	0.875
Spatial						
Stride Width M (cm.)	0.65	0.002*	0.60	0.011*	− 0.23	0.815
Temporal						
Stride Time M (sec.)	0.61	0.004*	0.57	0.016*	− 0.16	0.870
Variability						
Stride Length CV (cm.)	0.60	0.006*	0.36	0.039*	− 0.82	0.410
Stride Time CV (sec.)	0.68	< 0.001*	0.50	0.040*	− 0.76	0.448
Stance CV %	0.75	< 0.001*	0.57	0.016*	− 0.87	0.387
Total D. Support CV %	0.75	< 0.001*	0.66	0.004*	− 0.47	0.639
Stride velocity CV (cm./sec.)	0.73	< 0.001*	0.45	0.033*	− 1.16	0.248

**Table 3:** Mean (standard deviation), and [range] of the gait measures

Measure	Mean (SD) [range]
Pace	
Gait speed (cm/sec)	108.74 (34.13) [29.83, 147.74]
Rhythm	
Cadence (steps/min.)	106.84 (19.14) [59.64, 125.59]
Spatial	
Stride Width M (cm.)	13.29 (5.80) [4.67, 25.12]
Temporal	
Stride Time M (sec.)	1.23 (0.36) [0.95, 2.21]
Variability	
Stride Length CV (cm.)	8.24 (6.57) [1.96, 26.87]
Stride Time CV (sec.)	8.02 (7.13) [1.98, 25.05]
Stance CV %	4.92 (2.78) [1.69, 11.16]
Total D. Support CV %	9.75 (6.20) [3.42, 25.53]
Stride Velocity CV (cm./sec.)	8.47 (4.35) [3.06, 20.16]

Spearman’s rho is used to indicate correlation. Asterisks represent *P* < 0.05

*CV* coefficient of variance; *M* median. *P* P value. z Fisher *Z*

### Experiment 2

**Fig. 2:**
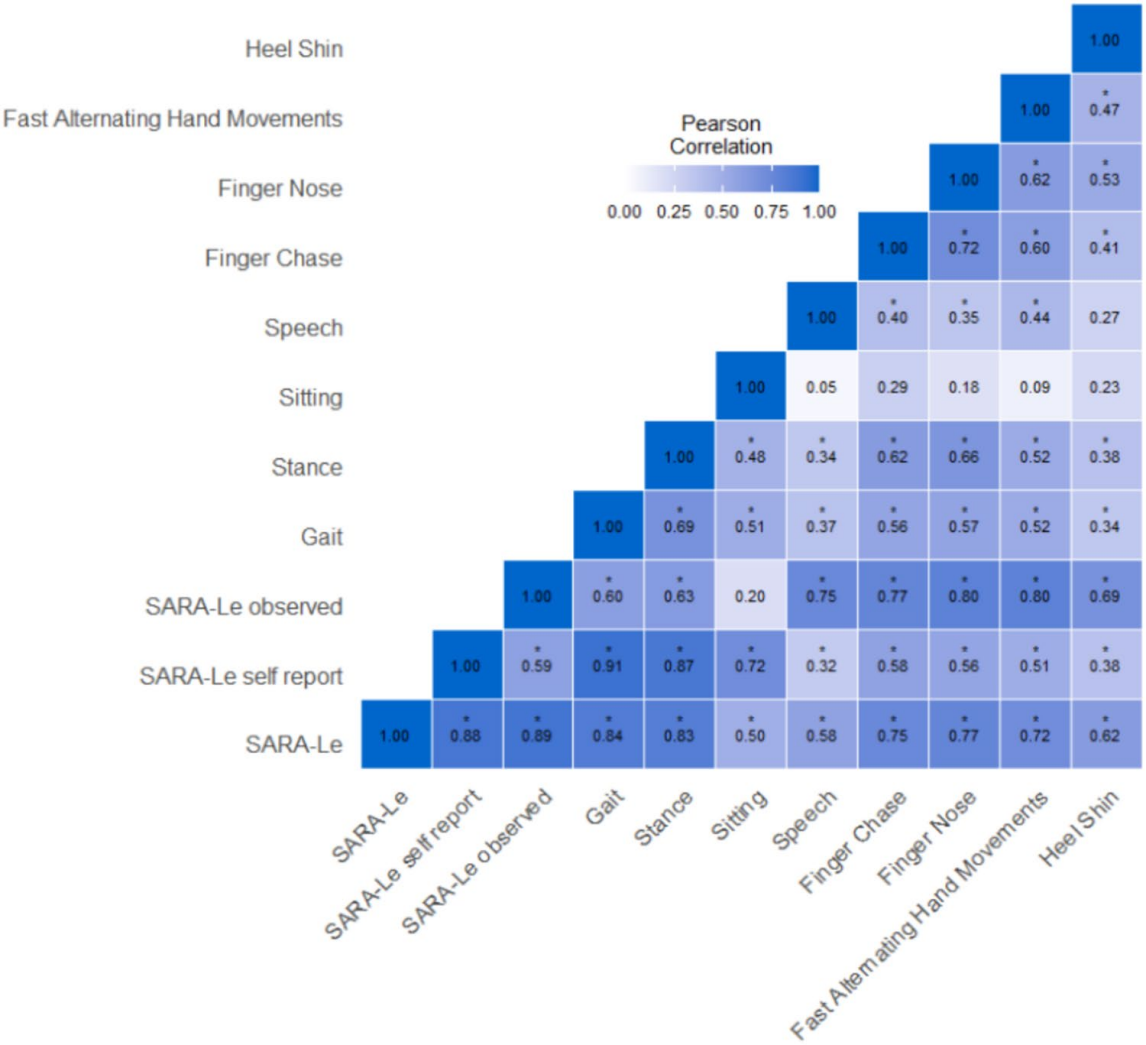
Correlations heatmap of all items in SARA-Le, including total score and the subsections (self-report and observed). Significant correlations are marked with an asterisk (*P* < 0.05).

**Fig. 3:**
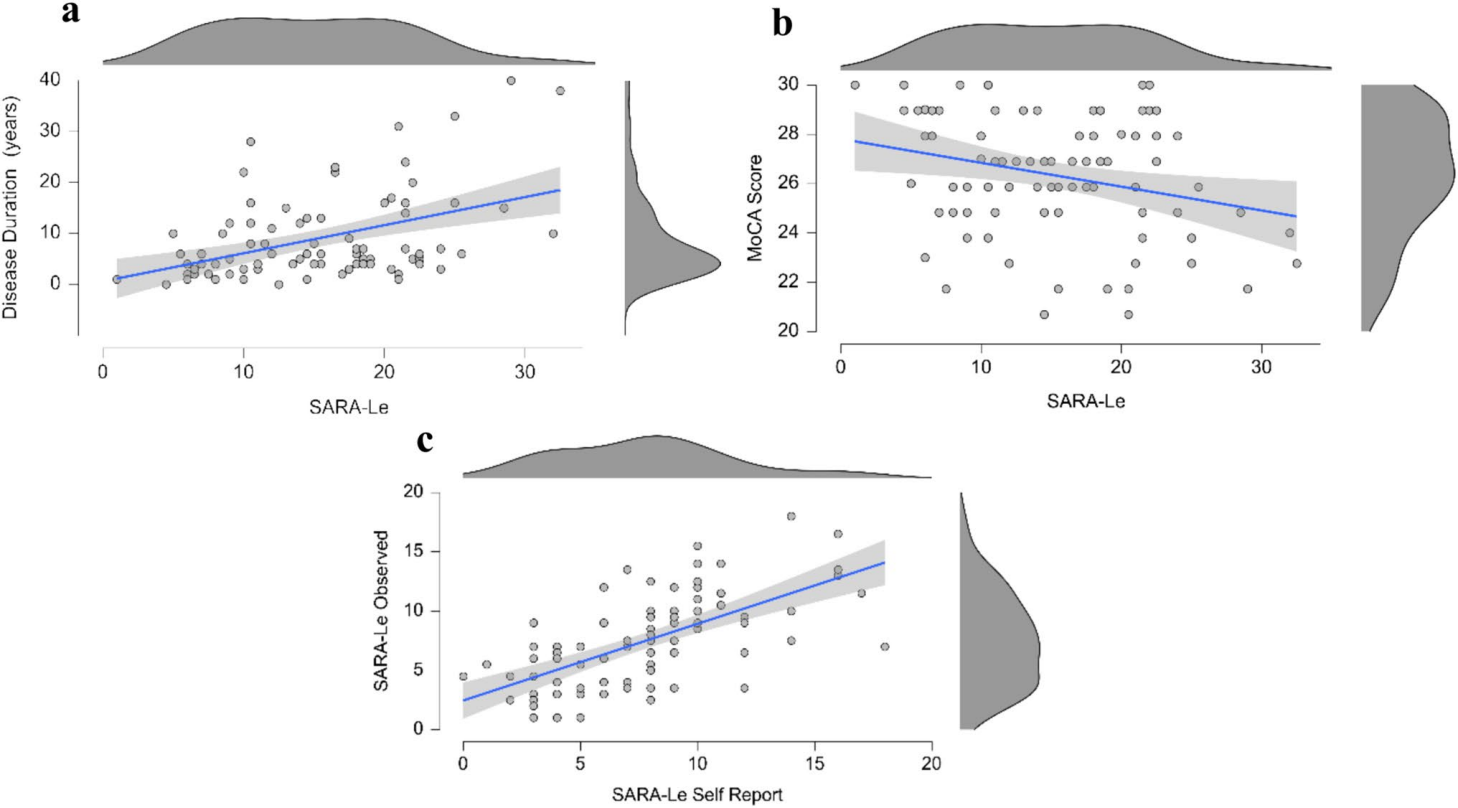
** a** Correlation between SARA-Le scores and disease duration. **b** Correlation between the SARALe and MoCA scores. **c** Correlation between the self-report and observed sections of the SARA-Le

The SARA-Le Cronbach’s Alpha value was high (0.831), demonstrating high internal consistency. Item-specific analysis correlation coefficients of the SARA-Le are shown below (Fig. 2). Additionally, in line with previous findings collected using the in-person SARA [18], a positive correlation was found between SARA-Le scores and disease duration (Fig. 3; *rho* = 0.39, *P* < 0.001), supporting construct validity. Second, the sum of the self-report section (questions 1–3) was highly correlated with the sum of the observed section (questions 4–8) of the SARA-Le (*r* = 0.63, *P* < 0.001). Although the first three items do not measure the same construct as the remaining items, this correlation indicates a strong association between the participants’ reports on the severity of the symptoms and the objective measures, suggesting internal consistency between subjective and objective measures. Though we cannot assess inter-rater reliability measures, we analyzed the mean differences across raters and found no significant discrepancies (*P* > 0.05). Third, consistent with existing in-person literature [19, 20], the SARA-Le scores were negatively correlated with MoCA scores (*r* = − 0.27, *P* = 0.007), supporting construct validity. Fourth, there was no significant correlation between the SARA-Le and age or years of education (*r* = 0.15, *P* = 0.18 and *r* = − 0.17, *P* = 0.11, respectively), supporting discriminant validity. Fifth, there was no difference in SARA-Le scores between genders (t(81) = 0.59, *P* = 0.55), supporting discriminant validity.

## Discussion

In the current study, we introduce SARA-Le, a video conferencing (VC) protocol specifically designed for the remote assessment of ataxia severity. This protocol, which is derived from the original in-person open-access Scale for the Assessment and Rating of Ataxia (SARA), offers both time and cost-effectiveness as it can be easily administered in the participant’s home environment using only a computer, internet connection, and camera.

We conducted a prospective validation study of SARA-Le involving patients with cerebellar ataxia (CA). The results demonstrated significant associations between SARA-Le and conventional measures. First, we found a very high positive association between the SARA-Le and SARA scores, supporting the convergent validity. Additionally, the ICC between the SARA and the SARA-Le was high, supporting reliability. Second, we observed significant correlations between SARA scores and nine spatial–temporal gait measures, which are typical characteristics of ataxic gait patterns. Consistent with these results, the SARA-Le scores also exhibited significant correlations with the nine gait measures, providing an independent aspect of construct validity. Third, the SARA-Le Cronbach's Alpha value was high, demonstrating high internal consistency. Fourth, in line with previous findings collected using the in-person SARA [16, 19, 20], a positive correlation was found between SARA-Le scores and disease duration, supporting construct validity. Fifth, consistent with existing in-person literature [17, 18], the SARA-Le scores were negatively correlated with MoCA scores, supporting construct validity. Taken together, the pattern of results underscores the feasibility and validity of SARA-Le. This converging evidence strengthens the case for the broader adoption of SARA-Le in both clinical and research settings.

To date, ataxia severity has been assessed mostly in-person. However, there have been attempts to adapt SARA for remote assessment through leveraging technology-based tools [9–11, 20–24]. Yet, most of these tools required the use of additional equipment such as an accelerometer [21] handheld video cameras [22], tablet devices [10], or devices from video games such as Microsoft Kinect, Leap Motion Controller [24]. Of the studies that previously examined the SARA through VC [9–11] there were several limitations. First, all studies that utilized VC were reliant upon a professional rater to assess the SARA score, which significantly reduces the scalability and accessibility as professionals typically have long waiting times and are an expensive resource. Second, some items from the SARA that required elements of balance were not modified through remote testing [11], introducing a safety concern. Finally, two of the studies had sample sizes of less than 20 participants [9, 11], which raises questions regarding the generalizability of these tools and the applicability of the findings to the general population. One of these [11] also exhibited a high dropout rate of 31.5% (6/19), which may further limit the representativeness of PwA.

The SARA-Le protocol does not require additional devices, or experts for administration or scoring, and can be safely administered at the participant’s home. The SARA-Le can be used either in conjunction with in-person SARA scores or as a standalone measure. In Experiment 2, we collected data from 83 CA participants, demonstrating the feasibility and scalability of SARA-Le. As the primary goals of Experiment 2 were to test feasibility and scalability, and since completing both in-person SARA and remote SARA-Le is time-consuming, the participants were administered only SARA-Le. Future research should extend Experiment 1 to a larger and more diverse CA cohort to evaluate generalizability.

While we have addressed some of the current challenges in the field, certain limitations need to be considered. One potential limitation of SARA-Le is its applicability to patients with severe motor or cognitive conditions. Motor or cognitive impairments could affect the participants’ understanding and response to the assessment procedures. Indeed, our study primarily involved participants without severe motor or cognitive impairment (MoCA > 21 and SARA < 22). It is important to note that other studies that utilized remote assessment of ataxia, such as the SARA^home^ [10], predominantly also included participants in the mild to moderate range of severity (SARA score < 20). Therefore, the findings of this study and others who have conducted remote evaluations should be interpreted with caution when applied to patient populations with severe motor or cognitive impairments. Future research should consider these factors and explore methods to accommodate patients with severe impairments to ensure the validity and applicability of the results to all ranges of disease severity.

One should consider unexplained noise in SARA-Le scores. In our investigation of the SARA-Le, we encountered a few factors that may introduce unexplained variability into the scores. These considerations are essential for understanding the robustness of this new online assessment tool. First, there is inter-rater variability. The SARA-Le, like its in-person counterpart, involves subjective assessments. The SARA is an inherently subjective test, lacking uniformity in both administering and scoring items [25]. Future research can investigate the inter-rater correlation between trained raters of SARA-Le and an expert neurologist clinician.

Second, the testing environment could have potential unexplained noise. Participants complete the SARA-Le remotely from their own homes. While this user-friendly approach is more convenient, it also introduces variability. The diverse testing environments—ranging from quiet bedrooms to noisy living rooms—may affect performance and contribute to score fluctuations. Third, three SARA-Le items (i.e., gait, stance, and sitting) rely on participants’ subjective evaluations of their abilities. To prioritize safety, we modified these items for online administration. However, this subjective self-assessment could potentially introduce additional variability into the scoring process. Despite potential noise, recent research [26] suggests that self-reported subjective items can effectively track disease progression in CA. Accordingly, we observed a strong correlation between self-reported scores and objective items in the SARA-Le. Fourth, the participants in Experiment 1 were limited to SCA3. Though this homogeneity allowed for a more precise evaluation of SARA-Le, future studies should include a more diverse range of disease severities and disease types. In summary, while acknowledging potential sources of unexplained noise, our current findings support the reliability and validity of the SARA-Le when compared to traditional in-person SARA assessments. Researchers and clinicians should consider these factors when interpreting scores and designing future studies.

This study confirms the feasibility, scalability, and validity of SARA-Le as a method for assessing ataxia severity remotely in PwA. Our remote VC test offers an efficient and convenient method of testing, making it accessible to a diverse population. SARA-Le addresses scalability and accessibility in-person testing challenges, permitting repeated monitoring and providing valuable insights into ataxia progression. Furthermore, the ability to monitor PwA frequently allows for more timely interventions. In an era emphasizing personalized medicine, remote testing holds promise for providing long-term monitoring in both motor and neuropsychological realms, particularly essential for rare neurodegenerative conditions. The SARA-Le is designed for both clinical and research applications to facilitate accessible and scalable assessment of ataxia severity. As we discussed, SARA-Le allows remote ataxia monitoring without requiring in-person visits, which can be particularly beneficial for longitudinal studies, clinical trials, and telemedicine applications. We hope that remote testing will be implemented in research and clinical settings, enhancing our understanding of ataxia progression and ultimately benefiting PwA worldwide.

## Footnotes

1 Subtypes were: 3 SCA1, 8 SCA2, 17 SCA3, 1 SCA5, 9 SCA6, 1 SCA7, 2 SCA8, 1 SCA10, 1 SCA17, 1 SCA28, 1 SCA42, 1 SCA44,1 SPG7, 1 ARSACS, 1 OPCA, 1 Sporadic, 1 Episodic, 1 Gluten, 2 Autoimmune, 2 Idiopathic, 1 Acquired (alcohol), 1 Perrault Syndrome, 1 Astrocytoma.
